# Supplementary material for: Causal Relationship between Aging and Anorexia Nervosa: A White-Matter-Microstructure-Mediated Mendelian Randomization Analysis
Source: Biomedicines. 2024 Aug 16;12(8):1874. doi: 10.3390/biomedicines12081874 (PMC11351342; doi:10.3390/biomedicines12081874)
Supplement: Supplementary file 1 [file biomedicines-12-01874-s001.zip › Supplementary Figure/Figure S4/Singe SNP plot of MR analysis for TL to ubm-b-1658.pdf]

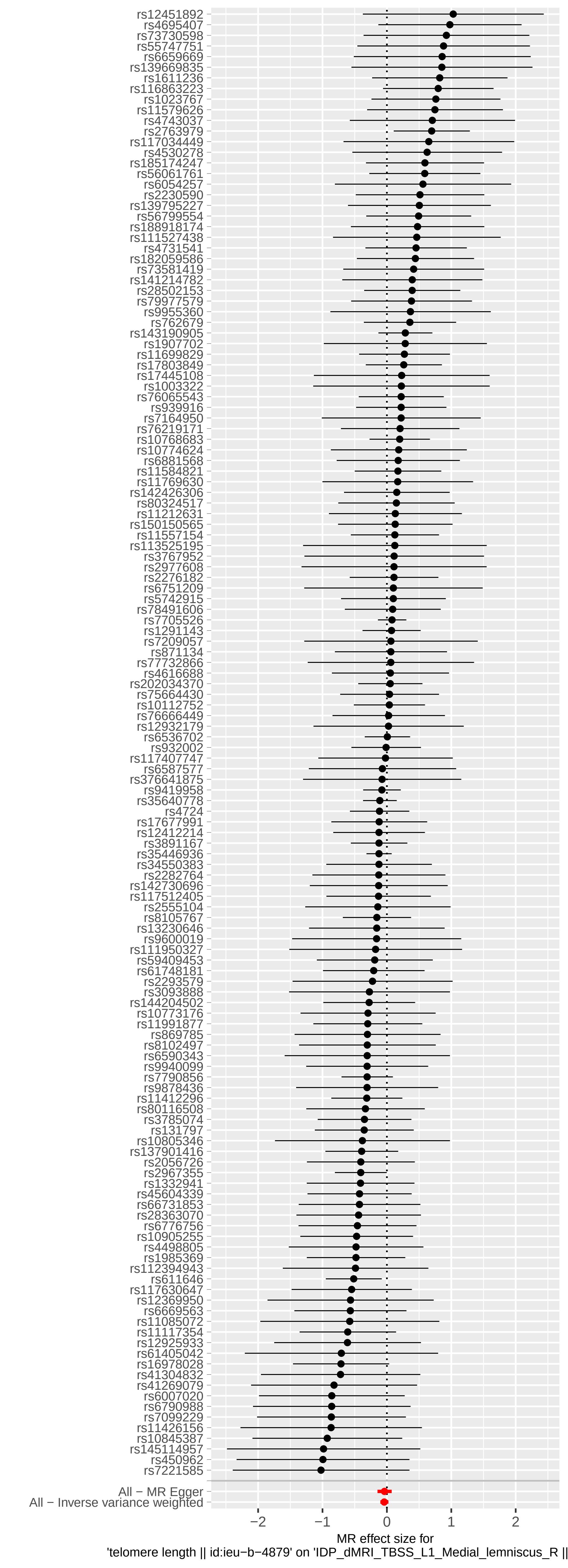
rs12451892
rs4695407
rs73730598
rs55747751
rs6659669
rs139669835
rs1611236
rs116863223
rs1023767
rs11579626
rs4743037
rs2763979
rs117034449
rs4530278
rs185174247
rs56061761
rs6054257
rs2230590
rs139795227
rs56799554
rs188918174
rs111527438
rs4731541
rs182059586
rs73581419
rs141214782
rs28502153
rs79977579
rs9955360
rs762679
rs143190905
rs1907702
rs11699829
rs17803849
rs17445108
rs1003322
rs76065543
rs939916
rs7164950
rs76219171
rs10768683
rs10774624
rs6881568
rs11584821
rs11769630
rs142426306
rs80324517
rs11212631
rs150150565
rs11557154
rs113525195
rs3767952
rs2977608
rs2276182
rs6751209
rs5742915
rs78491606
rs7705526
rs1291143
rs7209057
rs871134
rs77732866
rs4616688
rs202034370
rs75664430
rs10112752
rs76666449
rs12932179
rs6536702
rs932002
rs117407747
rs6587577
rs376641875
rs9419958
rs35640778
rs4724
rs17677991
rs12412214
rs3891167
rs35446936
rs34550383
rs2282764
rs142730696
rs117512405
rs2555104
rs8105767
rs13230646
rs9600019
rs111950327
rs59409453
rs61748181
rs2293579
rs3093888
rs144204502
rs10773176
rs11991877
rs869785
rs8102497
rs6590343
rs9940099
rs7790856
rs9878436
rs11412296
rs80116508
rs3785074
rs131797
rs10805346
rs137901416
rs2056726
rs2967355
rs1332941
rs45604339
rs66731853
rs28363070
rs6776756
rs10905255
rs4498805
rs1985369
rs112394943
rs611646
rs117630647
rs12369950
rs6669563
rs11085072
rs11117354
rs12925933
rs61405042
rs16978028
rs41304832
rs41269079
rs6007020
rs6790988
rs7099229
rs11426156
rs10845387
rs145114957
rs450962
rs7221585
All − MR Egger
All − Inverse variance weighted
−2
−1
0
1
2
MR effect size for
'telomere length || id:ieu−b−4879' on 'IDP_dMRI_TBSS_L1_Medial_lemniscus_R ||
